# Parasitological examination results of zoo animals in Germany between 2012 and 2022

**DOI:** 10.1016/j.ijppaw.2024.100942

**Published:** 2024-05-08

**Authors:** Lea-Christina Murnik, Ronald Schmäschke, Andreas Bernhard, Jens Thielebein, Klaus Eulenberger, Nadine Barownick, Sandra Gawlowska, Cora Delling

**Affiliations:** a Institute of Parasitology, Center for Infectious Disease, Faculty of Veterinary Medicine, Leipzig University, An Den Tierkliniken 35, 04103, Leipzig, Germany; b Zoo Leipzig, Pfaffendorfer Straße 29, 04109, Leipzig, Germany; c Institute of Agricultural and Nutritional Sciences, Martin Luther University Halle-Wittenberg, Theodor-Lieser-Straße 11, 06120, Halle (Saale), Germany; d Amerika-Tierpark Limbach-Oberfrohna, Tierparkstraße 1, 09212, Limbach-Oberfrohna, Germany

**Keywords:** Parasites, Zoo animals, Germany, Zoonotic disease

## Abstract

Parasitic infections in zoo animals are a critical concern for both animal health and management. The aim of this study was to assess the occurrence of endo- and ectoparasites among zoo animals in Germany. A retrospective analysis of the submitted samples of a diverse range of zoo animals (5768) from a ten-year period (2012–2022) was conducted. Overall, 31.1% of those samples tested positive for at least one parasite. In the examined samples, helminths (28.4%) were found more often than protozoans (10.3%) or ectoparasites (0.8%). Among the various animal groups the following parasites were found most commonly: Artiodactyla: Coccidia (34.6%), Strongylida (23.4%); Perissodactyla: Strongylida (19.3%), Ascaridida (12.0%); Carnivora: Ascaridida (16.6%), Coccidia (8.1%); Rodentia: Oxyurida (18.2%), Coccidia (10.5%); Marsupialia: Coccidia (9.4%), Oxyurida (5.9%); Primates: *Trichuris* spp. (9.7%), Oxyurida (2.2%); Aves: *Capillaria* (7.8%), Ascaridida (7.6%); Reptilia, Amphibia, Insecta: Oxyurida (18.7%); Pisces: Ciliates (6.2%). Furthermore, potentially zoonotic parasites were identified, including *Toxoplasma gondii* (0.1%), *Cryptosporidium* sp. (0.1%). By examining the occurrence of specific parasites, these findings demonstrate the importance of parasites in the context of zoo animal health. They also highlight the need for effective strategies to control parasite burden to improve the overall welfare of zoo animals.

## Highlights

• Overall prevalence of parasites in zoo animals from Germany was 31.1%.
• Helminths (28.4%) were found more often than protozoans (10.3%) or ectoparasites (0.8%).
• Potentially zoonotic parasites were identified (*Toxoplasma gondii, Cryptosporidium* spp.)

## Graphical abstract

**Figure:**
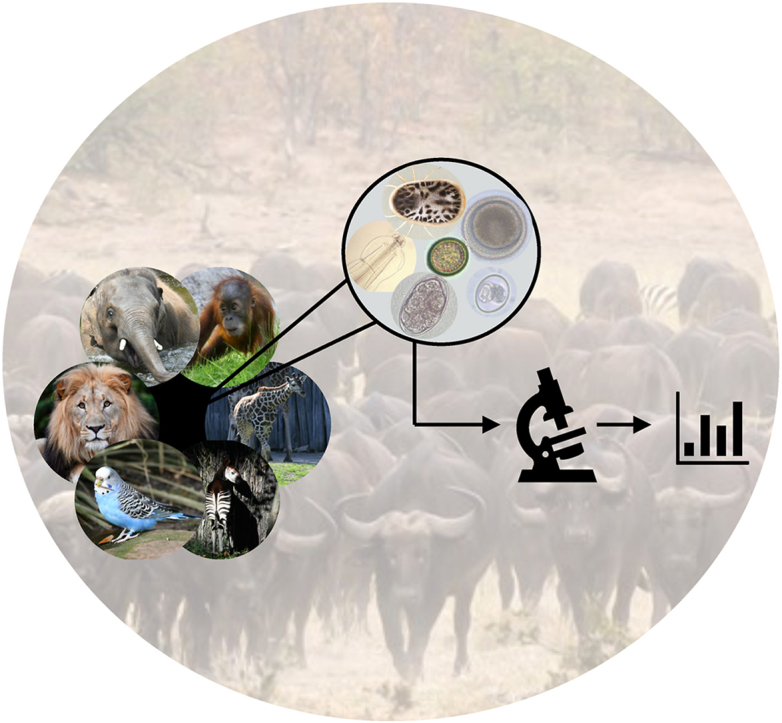

## 1 Introduction

Zoos and animal parks are popular tourist destinations and provide an excellent opportunity to observe wild animals and learn about their behaviors. In addition to providing educational opportunities, zoos also have a commitment to protect the biodiversity of animals and contribute to species conservation (Rose and Riley 2022), as they are involved in e.g., reintroduction programs or preservation breeding programs. Diagnosis and treatment of infectious diseases including parasitosis are essential in species conservation since parasites may cause severe disease and occasionally induce local reduction of the population size of endangered species (Cleaveland et al., 2002; Muoria et al., 2005). The living conditions in captivity confront zoos with challenges, particularly with regard to animal health (Cubas 1996).

The habitats of wild animals are usually quite large areas and include a high diversity of animal species, which may decrease the risk of an infection (Keesing et al., 2006). In addition, animals may develop natural protection against parasites creating a host-parasite balance in which the host suffers little or shows no clinical signs (Beck and Pantchev 2013). However, although enclosures of zoos and animal parks are normally designed to mimic the animals' natural habitats, the available space is restricted, and many different species live in close proximity to each other. Therefore, the zoo animals may be exposed to a potentially higher parasite load (Geraghty et al., 1981; Mbora and McPeek 2009; Mir et al., 2016). In these settings, diagnostics as well as control of parasitic infections are highly relevant for managing animal health. Furthermore, stressors such as space restriction or changes in social herd structures can affect the immune system making these animals perhaps more vulnerable for infections (Mbaya et al., 2009). Zoo animals may also be exposed to parasites not found in the animal's native habitat, such as the finding of *Taenia martis* in a lemur (Peters et al., 2023).

Additionally, zoo animals may harbor zoonotic parasites, such as *Toxoplasma gondii*, *Giardia* spp. Or *Trichuris* spp., and thereby, they are a potential source of infection for humans they are in close contact with, such as zookeepers. (Levecke et al., 2007; Denk et al., 2022).

Here, parasitological examinations of animals from zoos and animal parks in Germany over ten years were evaluated to gain a better understanding of the parasitic fauna.

## 2 Materials and methods

### 2.1 Samples

Between January 2012 and December 2022, a total of 5768 samples from zoos and animal parks were submitted to the Institute of Parasitology, Leipzig University for a parasitological examination. The samples originated from 41 different zoos and animal parks from all regions of Germany, but mainly from Eastern Germany. The reasons for diagnostic analysis were the conduction of a routine general health check or the transfer of animals to other zoos or health disorders. In addition, a parasitological examination of the intestinal tract was carried out when the animals were sent in for pathological examination.

Associated data regarding age, gender or clinical symptoms were only sporadically provided and could therefore unfortunately not be evaluated.

### 2.2 Examination of the samples

Depending on the respective question, the fecal samples were examined for the presence of parasites by different methods.

The most frequently used method was the combined flotation and sedimentation to detect endoparasites. Briefly, an approximately apricot-sized amount of feces was mixed with water, filtered, and then sedimented for 30 min. From the obtained sediment, 1 ml was poured into a test tube and topped with the flotation solution sodium nitrate (NaNO_3_, specific gravity of 1.3). After centrifugation (5 min, 2000 rpm) the obtained parasites were examined under the microscope (Schmäschke 2013a).

Another frequently used method was the parasitological examination of the gastrointestinal tract (GI). This involved scraping and rinsing the intestine to collect the contents. After filtering, GI contents were macroscopically examined for endoparasites.

To detect lungworms, the Baermann-Wetzel method was used to extract lungworm larvae from the feces (Deplazes et al., 2012).

The McMaster method, modified by Wetzel, was used for a quantitative analysis. A specific amount of feces (4g) was weighed, flotated (flotation solution sodium nitrate (NaNO_3_), specific gravity of 1.3) and eggs were counted using a McMaster chamber (Deplazes et al., 2012; Wetzel 1951). The threshold of this method is 50 eggs per gram of feces.

Molecular analysis was only used when specific parasites were suspected, and therefore, not conducted regularly. For the molecular detection of parasites different conventional PCR protocols were used depending on the respective parasite. *Cryptosporidium* spp. DNA was detected from fecal samples using a conventional PCR protocol targeting the 18S rRNA gene as previously described by Morgan et al. (1997). First, the fecal samples were treated with ultrasonics for 5 min. Afterwards, DNA was extracted from each sample using the QIAamp® Fast DNA Stool Mini Kit (QIAGEN, Hilden, Germany) according to the manufacturer's instruction. The reaction mixture contained 2.5 μl 10X DreamTaq Buffer (Thermo Scientific ™), 0.8 μl NTPs, 0.5 μl forward Primer (25 μM), 0.5 μl reverse Primer (25 μM), 0.1 μl DreamTaq Green DNA Polymerase (Thermo Scientific™), 3 μl of the DNA sample and DEPC water to a total volume of 25 μl.

To detect Toxoplasma gondii DNA, the B1 gene was targeted as previously described by Jalal et al. (2004). Samples were contained from pathological sectioning and were either fecal samples or organ samples e.g., brain or liver samples. The DNA from fecal samples was extracted as previously described, while DNA from organ samples was extracted using the NucleoSpin® Tissue Kit (MACHEREY-NAGEL, Düren, Germany) according to the manufacturer's instruction. The reaction mixture contained 2.5 μl 10X DreamTaq Buffer (Thermo Scientific ™), 0.8 μl NTPs, 0.4 μl forward Primer (25 μM), 0.4 μl reverse Primer (25 μM), 0.15 μl DreamTaq Green DNA Polymerase (Thermo Scientific™), 3 μl of the DNA sample and DEPC water to a total volume of 25 μl. Furthermore, the 18S rRNA gene was used to detect Apicomplexa DNA as previously described by Yang et al. (2001). The DNA was extracted as previously described. The reaction mixture contained 2.5 μl 10X DreamTaq Buffer (Thermo Scientific ™), 0.8 μl NTPs, 0.5 μl forward Primer (25 μM), 0.5 μl reverse Primer (25 μM), 0.2 μl DreamTaq Green DNA Polymerase (Thermo Scientific™), 2 μl of the DNA sample and DEPC water to a total volume of 25 μl. PCR products were visualized by gel electrophoresis using a 1.5% agarose gel stained with ethidium bromide. In case of a positive result sequencing was conducted for *Cryptosporidium* and Apicomplexa by Microsynth Seqlab (Göttingen, Germany). The obtained sequences were compared to sequences from GenBank® using the Basic Local Alignment Search Tool (BLAST) to identify the species.

For the diagnostic analysis of ectoparasites, skin samples, feather samples or the actual suspected ectoparasite were sent in. First, all samples were analyzed using a stereo microscope. Skin scrapings were additionally mixed with 10% potassium hydroxide solution (KOH). KOH was first heated to 70 °C, and then the skin scraping was added. After cooling to room temperature, the sample was examined under a microscope (Deplazes et al., 2012).

An overview of the methods used for parasitological examination is given in Table 1. Some of the samples were analyzed using more than one method, therefore, the total number of performed examinations is higher than the actual number of samples.

**Table 1:** Methods used for detecting parasites.

Method	Number of samples examined using the respective method (n)
Combined flotation and sedimentation	3541
Detection of Lungworms (Baermann-Wetzel)	601
Parasitological examination of GI	2139
Ectoparasite analysis	33
Species determination of endoparasites	15
Gill smears	16
PCR detection • *Toxoplasma gondii* (*T. gondii B1*-gene) • *Cryptosporidium* spp. (18S rRNA-gene) • Apicomplexa *(Isospora* spp.) (18S rRNA-gene)	16
	7
	7
	2
Quantitative detection (McMaster-method)	7
*Cryptosporidium-*Antigen detection (FASTest® CRYPTO Strip, MegaCor Diagnostik GmbH, Hörbranz, Austria)	1

### 2.3 Data analysis

The obtained data was collected in an Excel sheet and a descriptive frequency analysis was carried out using Microsoft Excel Version 16.71 (Microsoft Corporation, Redmond, USA) and SPSS statistics 27 (IBM, Armond, USA).

## 3 Results

In the 10-year time-period a total of 5768 samples were sent in for parasitological examination. Overall, 31.1% (1793/5768) of the samples tested positive for at least one parasite. Within the examined samples, helminths (1638/5768; 28.4%) were found more often than protozoans (593/5768; 10.3%) or ectoparasites (47/5768; 0.8%). Table 2 and Table 3 give an overview of the parasites found with their respective numbers and percentages.

**Table 2:** Endoparasites within the respective animal orders.

Parasites	Animal-Orders										
	Artiodactyla	Perissodactyla	Carnivora	Rodentia	Marsupialia	Primates	Aves	Reptilia	Amphibia	Pisces	others^a^
Strongylida	23.4% (203/868)	19.3% (32/166)	2.6% (19/728)	6.6% (27/411)	5.1% (13/254)	0.8% (4/497)	1.8% (38/2088)	4.0% (20/496)	5.9% (2/34)	–	2.9% (4/139)
*Capillaria*	8.6% (75/868)	–	5.1% (37/728)	2.7% (11/411)	2.8% (7/254)	0.2% (1/497)	7.8% (162/2088)	0.4% (2/496)	–	2.5% (2/81)	5.0% (7/139)
Oxyurida	0.3% (3/868)	–	0.7% (5/728)	18.2% (75/411)	5.9% (15/254)	2.2% (11/497)	0.1% (1/2088)	18.7% (93/496)	–	–	2.2% (3/139)
Ascaridida	2.0% (17/868)	12.0% (20/166)	16.6% (121/728)	1.0% (4/411)	–	1.6% (8/497)	7.6% (158/2088)	5.2% (26/496)	–	–	4.3% (6/139)
*Trichuris*	16.5% (143/868)	–	1.1% (8/728)	6.8% (28/411)	1.2% (3/254)	9.7% (48/497)	0.1% (1/2088)	–	–	–	–
Hookworms	–	–	0.4% (3/728)	–	–	–	–	0.6% (3/496)	–	–	–
Spirurida	–	–	0.1% (1/728)	–	–	–	0.2% (4/2088)	–	–	–	–
*Trichosomoides*	–	–	–	0.1% (1/411)	–	–	–	–	–	–	–
*Strongyloides*	0.7% (6/868)	–	0.7% (5/728)	0.7% (3/411)	0.8% (2/254)	1.2% (6/497)	0.1% (2/2088)	0.6% (3/496)	–	–	1.4% (2/139)
*Setaria*	0.1% (1/868)	–	–	–	–	–	–	–	–	–	–
Lungworms	2.8% (24/868)	–	–	–	–	–	–	0.1% (1/496)	–	–	2.2% (3/139)
Cestoda	1.0% (9/868)	–	1.0% (7/728)	5.1% (21/411)	4.3% (11/254)	0.6% (3/497)	1.5% (31/2088)	1.6% (8/496)	–	–	–
Trematoda	0.1% (1/868)	–	–	–	–	–	0.4% (8/2088)	0.1% (1/496)	–	–	–
Coccidia	34.6% (300/868)	–	8.1% (59/728)	10.5% (43/411)	9.4% (24/254)	0.2% (1/497)	6.4% (134/2088)	4.0% (20/296)	–	–	2.2% (3/139)
*Cryptosporidium* spp.	0.2% (2/868)	–	–	–	–	–	–	0.1% (1/496)	–	–	0.7% (1/139)
*Toxoplasma gondii*	–	–	0.3% (2/728)	–	–	–	0.1% (1/2088)	–	–	–	–
*Neospora caninum*	–	0.6% (1/166)	–	–	–	–	–	–	–	–	–
*Balantidium*	–	–	–	–	–	–	–	0.1% (1/496)	–	–	–
*Acanthocephala*	–	–	–	–	–	–	0.2% (3/2088)	–	–	–	–
*Gyrodactylus* sp.	–	–	–	–	–	–	–	–	–	1.2% (1/81)	–
Pentastomida	–	–	–	–	–	–	–	0.1% (1/496)	–	–	–
*Ciliate*	–	–	–	–	–	–	–	–	–	6.2% (5/81)	–

a = including animals (n) of the order: Proboscidea (8), Pilosa (22), Macroscelidea (36), Pholidota (10), Cingulata (28), Eulipotyphla (4), Chiroptera (30).

**Table 3:** Ectoparasites within the respective animal group.

Parasites	Animal-Groups						
	Artiodactyla	Carnivora	Rodentia	Marsupialia	Primates	Aves	Others^a^
Mite	–	–	3.4% (14/411)	0.8% (2/254)	0.6% (3/497)	0.3% (6/2088)	0.7% (1/139)
Ticks	0.1% (1/868)	–	–	–	–	0.1% (2/2088)	–
*Trichodectidae*	0.9% (8/868)	–	0.1% (1/411)	0.4% (1/254)	–	–	–
Flea	–	0.1% (1/728)	1.0% (4/411)	–	0.2% (1/497)	–	–
Lice	–	–	–	–	–	–	1.4% (2/139)

a = including animals (n) of the order: Proboscidea (8), Pilosa (22), Macroscelidea (36), Pholidota (10), Cingulata (28), Eulipotyphla (4), Chiroptera (30).

### 3.1 Endoparasites

#### 3.1.1 Artiodactyla and Perissodactyla

In the 868 examined Artiodactyla samples the most prevalent parasites were coccidia (*Eimeria* spp.) (34.6%) which was mainly found among Moschidae (55.9%) and Bovidae (49.3%) (Supplementary Material Table 1), followed by Strongylida (23.4%) and *Trichuris* spp. (16.5%). In two samples of juvenile animals (dall sheep and dwarf goat) the potentially zoonotic protozoan *Cryptosporidium* spp. was identified. Other parasites detected are shown in Table 2.

In the order Perissodactyla (n = 166 samples) only three different parasites were detected (Table 2), whereby Strongylida (19.3%) was the most frequently found parasite, followed by Ascaridida (12.0%). All roundworm eggs were *Parascaris* spp. Only Equidae were found to have both of these parasites (Supplementary Material Table 1).

#### 3.1.2 Carnivora

A total of 728 samples were from the order Carnivora. Ascaridida eggs (16.6%) were diagnosed most frequently, including species from the genera *Toxocara* spp. (53.7%), *Toxascaris* spp. (27.3%) and *Baylisascaris* spp. (17.4%). Two of the samples (1.6%) did not include further information about the detected species. Ascaridida eggs were most commonly found among the family Felidae, especially Felinae (35.7%) were infected with ascarids (Supplementary Material Table 1). The second most prevalent parasite found were coccidia oocysts, which were detected in 8.1% of the samples; all coccidia oocysts were identified as *Cystoisospora* spp. and were found mainly in mongooses (28.6%) (Supplementary Material Table 1). Oocysts of the zoonotic parasite *Toxoplasma gondii* were detected in two samples (0.3%) from mongoose. Further information of parasites found within the samples of the Carnivora are shown in Table 2.

#### 3.1.3 Rodentia

In the order Rodentia (n = 411 samples) nematodes from the order Oxyurida were found most frequently (18.2%). They were detected mainly in Myomorpha (38.1%). Further information about the species present was documented only in 25 of the 75 positive samples. The following Oxyurida species were detected: *Syphacia* spp. (24%), *Aspiculuris tetraptera* (8%) and *Passalurus ambiguous* (5.3%). Furthermore, coccidia were detected in 10.5% of the samples. Especially Lagomorpha (60%) excreted coccidia oocysts. Parasites found in addition to those are shown in Table 2.

#### 3.1.4 Marsupialia

From the order Marsupialia 254 samples were sent in*.* Coccidia oocysts were found most frequently with 9.4% of the samples being positive, followed by Oxyurida (5.9%), Strongylida (5.1%) and Cestoda (4.3%). Additional parasites were found at small percentages (Table 2).

#### 3.1.5 Primates

**Fig. 1:**
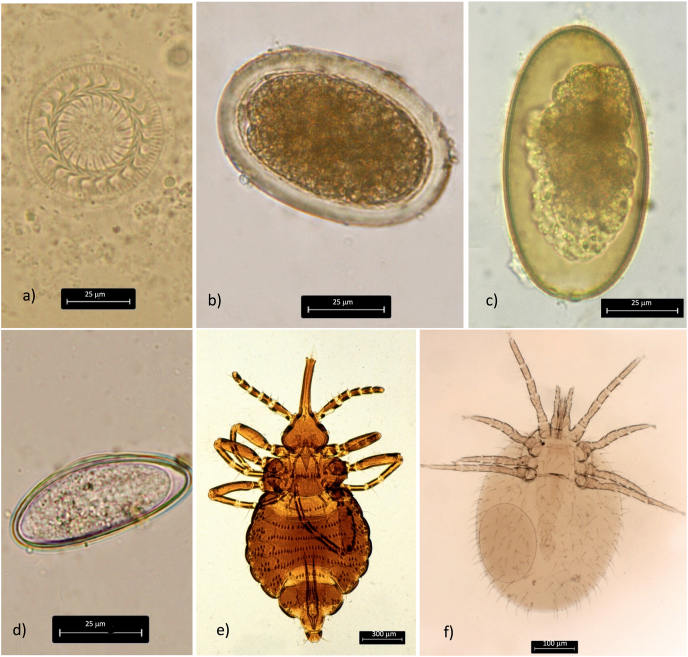
a) *Trichodina* spp. from a koi; b) Egg of *Ascaridia platyceri* from a *Platycercus elegans*; c) Egg of Oxyurida from a turtle; d) Egg of *Enterobius vermicularis* from a chimpanzee; e) *Haematomyzus elephantis* from an elephant; f) *Ornithonyssus bacoti* from a hamster.

A total of 497 samples from Primates were sent in for examination*.* The samples belong to four different groups of primates: Prosimian (n = 49), Catarrhini (n = 159), New World monkeys (n = 144) and Hominidae (n = 145). *Trichuris* spp. was diagnosed most frequently (9.7%), especially within samples of Catarrhini (28.9%), followed by Oxyurida (2.2%) (Fig. 1d), Ascaridida (1.6%) and *Strongyloides* (1.2%). In addition, other parasites were diagnosed at low percentages (Table 2).

#### 3.1.6 Aves

Overall, 2088 samples belonging to different bird species were examined. The most frequently found parasite was *Capillaria* in 7.8% of the samples. Furthermore, 7.6% of the samples tested positive for Ascaridida. All of the respective samples belonged to the two species *Ascaridia* spp. (Fig. 1b) and *Heterakis* spp., which eggs cannot be distinguished easily from each other using the light microscope. Birds from the orders Psittaciformes (15.2%; 16.8%), Galliformes (16.8%; 24.8%) and Accipitriformes (25%; 37.5%) were infected with the *Capillaria* and Ascarids respectively (Supplementary Material Table 1). Furthermore, coccidia oocysts were detected in 6.4% of the samples especially in the orders Passeriformes (17.6%) and Galliformes (14.7%). The zoonotic protozoan *T. gondii* was also found within Aves*.* After pathological examination, samples of the brain, liver and spleen of a western crowned pigeon were analyzed by PCR and found positive for *T. gondii*. Further parasites occurring in this order are listed in Table 2.

#### 3.1.7 Reptilia, Amphibia, insecta

In total, 496 reptile samples were analyzed. Most of the parasites detected were from the order Oxyurida (Fig. 1c), which was diagnosed in 18.7% of the samples. Testudinata were infected most frequently (23%). Furthermore, we were also able to identify the protozoan *Cryptosporidium* in one sample (Table 2).

A few samples belonged to the order Amphibia (n = 34) and Insecta (n = 6). Only Strongylida (5.9%) were identified within the order Amphibia. No parasites were found within the samples from insects.

Specific species of the parasites found within the orders Reptilia and Amphibia were not determined.

#### 3.1.8 Pisces

Of the 81 examined fish samples, a number of different parasites could be identified. Ciliates were found in 6.2% of the samples. Ciliata samples were either *Ichthyophthirius multifiliis* (40%) or *Trichodina* spp. (40%) (Figs. 1a) and 20% were *Glossatella* sp. Furthermore, *Capillaria* spp. and *Gyrodactylus* spp. were identified in 2.5% and 1.2% of the samples respectively (Table 2).

#### 3.1.9 Others

Due to small sample numbers the following animal groups were combined and summarized: Proboscidea (n = 8), Pilosa (n = 22), Macroscelidea (n = 36), Pholidota (n = 10), Cingulata (n = 28), Eulipotyphla (n = 4), Chiroptera (n = 30).

The parasite occurrence within this group was evenly distributed. *Capillaria* eggs were detected most frequently (5.0%). Eulipotyphla (60%) and Pilosa (18.2%) were infected most commonly. In addition, in one sample from a hedgehog *Cryptosporidium* spp was identified. Additional information about the occurring parasites is given in Table 2.

### 3.2 Ectoparasites

Only a small number of ectoparasites or samples were sent in for identification and analysis, so the ectoparasite results should be considered carefully. Ectoparasites were present in 0.8% of the overall samples (Table 3).

Animals of the orders Artiodactyla and Perissodactyla were mainly infected with Trichodectidae (n = 8). Of these parasites seven were assigned to *Bovicola* sp., whereas one was not further specified. Furthermore, one tick was found and identified as *Ixodes ricinus.*

Within Carnivora only one positive sample from a maned wolf was found and was identified as *Ctenocephalides felis*.

Animals from the order Rodentia showed the most positive results of ectoparasites. Mites (n = 14) were found predominantly among rodents followed by fleas (n = 4). All mites were identified as *Myocoptes musculinus*, which is known to occur in mice. Three of the respective fleas were identified as *Xenopsylla cheopis* and one was assigned to the species *Monopsyllus sciurorum.* Moreover, one chewing louse was identified from a guinea pig which belonged to the species *Gliricola porcelli.*

Within Marsupialia one ectoparasite of the family Trichodactylidae was identified from a wallaby. Furthermore, two mites could be identified from two kowaris both belonging to the species *M. musculinus.*

From Primates, three mites were identified as *Demodex* sp. and one flea was identified as *C. felis*.

Within Aves predominately mites (n = 6) were found which belonged to different feather mite species (*Ptiloxenoides phoenicopteri*, *Pterophagus* sp.) and one sample was assigned to *Ornithonyssus sylviarum.* Moreover, two ticks were found, both identified as *I. ricinus.*

In the remaining orders one mite (*Ornithonyssus bacoti*) (Fig. 1f) was found in a sample from a round-eared elephant shrew. Furthermore, two lice (*Haematomyzus elephantis*) (Fig. 1e) were identified in samples from elephants.

## 4 Discussion

This study provides an overview of the parasite fauna in various animal species from several German zoos and animal parks. Overall, the positivity rate in the present study was 31.1%. In comparison with previous studies the obtained occurrence rate is rather low. For example, in Brazil at the Rio de Janeiro Zoo the overall prevalence rate was estimated as 68.3% (Barbosa et al., 2020). In two zoological gardens in Italy the overall prevalence was 61.5% (Fagiolini et al., 2010). In Malaysia an overall prevalence of 56.3% was estimated (Lim et al., 2008). However, comparing studies from different countries is rather difficult due to different environmental and climatic conditions, which may affect the survival of infectious parasite stages in the environment (Barbosa et al., 2020).

In this study helminth infections (28.4%) were more often identified than protozoal infections (10.3%). This is in accordance with previous studies conducted in zoos (Lim et al., 2008; Fagiolini et al., 2010; Mir et al., 2016). However, this observation is contrary to the studies of Pérez Cordón et al. (2008) and Levecke et al. (2007), where protozoans were detected more frequently than helminths. It has been suggested that the simplicity of their lifecycle, as they do not need an intermediate host and some of them are immediately infective after excretion, contributes to a parasitic infection. Moreover, the high environmental tenacity of excreted stages, low infection doses and short prepatent periods make transmission very effective, especially in confined spaces (Levecke et al., 2007; Dhakal et al., 2023). This may also be the case for the parasites found in this study, as the protozoans (e.g., coccidia*, Cryptosporidium* spp.) and the most commonly found helminths (*Strongyloides, Capillaria,* Oxyurida, Ascaridida*, Trichuris*) have a simple life cycle without an intermediate host, making transmission easy in a confined environment (Schnieder 2006).

Considering all types of parasites found, coccidia were detected most frequently (10.3%). This is in accordance with a previous study conducted in a zoo in Spain, in which 432 samples were investigated over a time period of one year (Pérez Cordón et al., 2008). Here, coccidia were detected in almost all animal groups except three (Perissodactyla, Amphibia and Pisces). Although we did not find coccidia, all three groups could also be infected with those parasites: *Eimeria leuckarti* is known to infect Equidae within the order Perissodactyla*.* Amphibia can be infected by multiple species of *Eimeria, Isospora* and *Goussia*, and in Pisces several species of *Eimeria* and also *Goussia* may occur (Schnieder 2006). However, in this study, coccidia were detected most frequently in the order Artiodactyla (34.6%) followed by Rodentia (10.5%), Marsupialia (9.4%) and Carnivora (8.1%) which is in accordance with Dhakal et al. (2023) and Pérez Cordón et al. (2008) who also found coccidia most prevalent in Artiodactyla at 34.28% and 31.8%, respectively. In our study, ruminants (Moschidae and Bovidae) were most frequently infected with coccidia. It is known that different *Eimeria* species in several animals vary in their pathogenicity. Regarding domestic animals (e.g., cattle), several species of *Eimeria* can parasitize in the GI, but two species (*E. zuernii* and *E. bovis*) are known to be pathogenic especially in calves and young cattle (Daugschies and Najdrowski 2005). Infection may cause severe diarrhea, dehydration, anorexia and even death, particularly in young animals. Therefore, parasite screening and, if possible, analysis of the occurring species is necessary to ensure the animals’ health.

Parasites of the family Strongylidae were detected in 6.3% of the overall samples, and mainly found in Artiodactyla (23.4%) and Perissodactyla (19.3%). This is in accordance with previous studies (Lim et al., 2008; Fagiolini et al., 2010). Strongylids are common parasites of ruminants and horses. Depending on the severity of the infection, strongylids may cause significant clinical disease, such as anemia and hypoalbuminemia caused by *Haemonchus contortus* in small ruminants (Carson et al., 2023). Furthermore, Equidae (horses, zebras, donkeys, mules) are regularly affected by strongylids, as shown in a study on Przewalski's horses, in which strongylids were detected most often (Jota Baptista et al., 2021). Moreover, *Trichostrongylus* sp. and *H. contortus* were the most frequently found parasites with 78% and 55% respectively in Grevy's zebras in Kenya (Muoria et al., 2005). Regarding the clinical impact strongylids may have in terms of e.g., colic or larval migration, and widely spread anthelmintic resistances exist (Reinemeyer and Nielsen 2009), those parasites are of great importance, especially concerning the reduction of the parasitic burden.

Furthermore, Ascarididae species were identified in 6.2% of the samples. The most frequently affected animal groups were Carnivora (16.6%), Perissodactyla (12.0%) and Aves (7.6%). Another study from two zoos in Italy showed similar results, but they found even higher detection rates of an ascarid infection especially in felids (66.7%) (Fagiolini et al., 2010). This is in line with our results, since within the order of Carnivora, felids were most frequently infected with ascarids. It has been known that *Toxocara* spp. is one of the most common roundworms in Felidae as well as in Canidae (Lim et al., 2008). *Toxocara* spp. and *Toxascaris* spp. have been described as commonly occurring nematodes in big cats in the wild as well as in zoo animals (Muller-Graf 1995; González et al., 2007; Schieber and Štrkolcová 2019). It seems that especially big cats (Pantherinae) such as lions or tigers but also the smaller cats (Felinae) like cheetahs are often infected (Lim et al., 2008; Fagiolini et al., 2010). The clinical impact of an ascarid infection depends on the parasitic load, but is usually associated with minimal to mild symptoms e.g., diarrhea and weight loss (Deplazes et al., 2012). A pathological study on jungle cats also showed only mild intestinal lesions, although 86% of the cats were infected with *T. cati* (Tabaripour et al., 2018). Moreover, *Toxocara* spp. has a zoonotic potential and infection in humans may clinically manifest in form of larva migrans visceralis (Schieber and Štrkolcová 2019). Therefore, measures to control ascarids in zoo animals seem to be reasonable in sense of the One Health concept.

The high occurrence of coccidia, strongyles and parasites of the family Ascarididae in Artiodactyla, Perissodactyla and Carnivora is not unexpected considering their common occurrence in domestic animals such as horses, cattle, dogs or cats (Barutzki and Schaper 2011; Rehbein et al., 2013; Carminatti et al., 2023). These animals have comparable behaviors, such as grazing, feeding and social interaction, leading to similar parasite transmission routes. Also, the living conditions of domestic animals, often characterized by shared spaces, exposure to environmental contaminants and the proximity to other animals, are very similar to those of zoo animals.

It should be noted that some of the herein detected parasites may not have actually infected the intestine of the examined animal, but passed through as a result of consuming infected food. This phenomenon, known as pseudoparasitism, is particularly common in reptiles, especially snakes, and marsupials such as the quoll, but may also occur in carnivores. Food animals (e.g., mice) may be infected with coccidia or oxyurids whose parasites then be mistaken for resident parasites in the examined reptiles, snakes or marsupials (Schmäschke 2013b). Therefore, the high levels of oxyurids and coccidia observed in these groups should be interpreted with caution, given the challenges associated with accurate morphological species identification of these parasites, which can be often difficult or even impossible (Schmäschke 2013b).

Of particular interest was the finding of *Toxoplasma gondii* in two samples of carnivores and one avian sample. All three samples were diagnosed using PCR and *T. gondii* was found in the brain of the two meerkats and in the brain, liver and spleen of the crowned pigeon sample. Several studies have detected *T. gondii* in zoo animals (Bártová et al., 2018; Cano-Terriza et al., 2020; Denk et al., 2022; Liu et al., 2022). Toxoplasmosis is often clinically asymptomatic in intermediate hosts, but in some cases, it may cause symptoms, particularly in kangaroos or new world porcupines, where severe clinical signs such as ataxia, dyspnea and weight loss may occur (Beck and Pantchev 2013). Furthermore, meerkats, among others, seem to belong to the clinically most affected animals related to *T. gondii* (Burger et al., 2017; Denk et al., 2022). In this study, we do not know where the infections took place since not all animals were born in the place where they lived finally. However, there are several transmission routes possible. Meerkats are occasionally fed with small mammals (e.g., mice), which can act as intermediate hosts within the life cycle of *T. gondii* (Deplazes et al., 2012). However, no investigations on the occurrence of *T. gondii* stages in food animals were made in this study. Moreover, it has been shown that also fruits and vegetables can be a source for infection (Pleyer et al., 2019). Furthermore, it has to be considered that cats (strayed and owned) may access zoo areas and contaminate the environment by shedding oocysts (Deplazes et al., 2012). But this route of transmission can be regarded as rather unlikely as studies on stray cats and owned cats from Germany determined only very low prevalences (0.1% and 0.8% respectively) of *T. gondii* (Barutzki and Scharper 2011; Becker et al., 2012). However, it has to be kept in mind that the occurrence of *T. gondii* in this study may be underestimated since no serological testing was performed regularly, and therefore, the actual occurrence might be higher. *T. gondii* has zoonotic potential and may therefore also be a risk for people working in close contact to animals (Tenter et al., 2000; Adjemian et al., 2012; Forsyth et al., 2012). In a previous study, zoo staff and zoo animals were screened for zoonotic infections, and T. gondii was demonstrated to occur among zoo animals as well as the staff. Nevertheless, the prevalence among the staff members did not differ much in comparison to the previously known local seroprevalence. Therefore, the risk of a potential transmission was estimated to be rather low. However, authors concluded that precautions should be taken (Forsyth et al., 2012). A case control study in Mexico could not find an association between human *T. gondii* infection and the exposure to animals (Alvarado-Esquivel et al., 2014). Further studies may clarify the potential zoonotic risk.

Furthermore, potential zoonotic *Cryptosporidium* spp. were identified using an antigen ELISA, PCR or staining (Heine-staining) in two juvenile Artiodactyla (dall sheep and dwarf goat) samples, one sample of a reptile, which belonged to the species *C. avium* and one sample of a hedgehog. The frequency of *Cryptosporidium* spp. might also be underestimated, since not all samples were screened for *Cryptosporidium* routinely. But the rather low occurrence of *Cryptosporidium* is in accordance with previous studies (Matsubayashi et al., 2005; Barbosa et al., 2020; Karim et al., 2021). Two studies focused on the occurrence of *Cryptosporidium* in Asian zoos estimated a low prevalence in the examined animals (0.4% and 3.5%) (Matsubayashi et al., 2005; Karim et al., 2021).

In this study, only 0.8% of the samples were positive for ectoparasites. However, this result must take into account that only a few feather, skin, hair samples or the actual parasite were sent in for parasitological analysis. Additionally, it has to be kept in mind that if animals showed similar symptoms after the diagnosis of a parasitosis and following treatment, the animals were usually treated again without any further examination. Unfortunately, we do not have any information about clinical symptoms shown by the infected animals.

Studies on the occurrence of ectoparasites in zoo animals are very limited with only one other published study from Nigeria (Tags et al., 2020). The parasite occurrence found in the respective study was rather low as they only found two of 33 samples being positive for ectoparasites (one tick, *Amblyomma marmoreum*, and one mite, *Sarcoptes scabiei*) (Tags et al., 2020). In our study, mites were the most commonly detected ectoparasites. Mainly found in mice and belonging to the species *Myocoptes musculinus*, which is well described in feeding and laboratory mice (Beck and Pantchev 2013). Moreover, mites were also found frequently in birds, whereby feather mites (e.g., *Ptiloxenoides phoenicopterid*) were found most commonly. Studies of native birds (e.g., finches, tits, woodpeckers) have shown that a large proportion (56.12% of 5071 samples) are infected with feather mites (Schöne and Schmäschke 2011). Therefore, it is not surprising that these mites are also increasingly found in zoo animals, since outdoor housing of birds may provide direct contact with wild birds and lead to a potential transmission of the pathogen.

Chewing lice (*Bovicola* sp.) were detected quite frequently in samples from ungulates which is comparable to the occurrence in domestic animals. In a study from Ireland on 51% of 652 examined animals chewing lice were found, whereby 88% of those cattle were infected with *Bovicola bovis* (Mckiernan et al., 2021). Ticks found in this study were identified as *I. ricinus*, one of the most common ticks in Germany (Rubel et al., 2023). Since ectoparasites may play an important role as vectors for multiple diseases (Nelder et al., 2009), further studies of their occurrence in wild animals should be performed.

The occurrence of parasites may be related to the type of enclosure (i.e., indoor or outdoor) and the number of animals living in an enclosure, as well as individual animal features such as age and immune status. The enclosures of the zoos considered here vary greatly according to the species being kept, nevertheless, great attention is paid to mimic the natural habitats of the individual species. Among other things, clinical relevance depends on the individual parasitic burden and the individual health status, as not all infections lead to serious health problems. It is difficult to summarize all hygienic procedures conducted in the included zoos and animal parks since they differ greatly between zoos and from one enclosure to another. Hygienic practices that are performed exemplarily in one of the included zoos are the change of bedding as well as removing the feces daily. In addition, food is offered in feeding troughs to avoid contact with the ground. Nevertheless, the conduction of hygienic procedures in open-air enclosures is often difficult and therefore, especially those enclosures are ideal reservoirs for parasites. Some parasitic stages (e.g., *Cryptosporidium*-oocysts, roundworm eggs) are highly resistant in the environment and may survive for months in a cold and damp environment, and so, preventing infections may not be possible (Deplazes et al., 2012; Barbosa et al., 2020). Therefore, the parasitic burden of animals from zoos should be controlled regularly to ensure good health conditions of the individual as well as to minimize environmental contamination and the infection risk for the whole population.

## 5 Conclusion

In conclusion, the importance of monitoring parasites in zoos cannot be overemphasized. Parasite stages were found in various animal species analyzed in this study. Potentially zoonotic parasites (e.g., *Toxoplasma gondii, Cryptosporidium* sp.) were identified. Our findings highlight the need for regular parasite screening in zoos and animal parks to minimize the contamination of enclosures and reduce the parasitic burden on animals. This approach is in line with the principles of a One Health concept, ensuring optimal health care for both, humans and animals.

## Funding

Funded by the Open Access Publishing Fund of Leipzig University supported by the German Research Foundation within the program Open Access Publication Funding.

## Authors’ contributions

All Authors contributed to the study conception and design. Lea Murnik: Analysis of the data, Visualization, Writing -original draft. Cora Delling: Conceptualization, Writing -original draft, Supervision. Ronald Schmäschke: Writing -review and editing. Andreas Bernhard: Sample submission, writing -review and editing. Jens Thielebein: Sample submission, writing -review and editing. Klaus Eulenberger: Sample submission, writing -review and editing. Nadine Barownick: Analysis of the samples. Sandra Gawlowska: Analysis of the samples.

## Ethics approval

Not applicable.

## Consent for publication

All authors agreed to the publication of the manuscript.

## Declaration of competing interest

The authors declare that they have no conflicts of interest. All authors agree to the submission of the manuscript.

## Appendix A Supplementary data

Multimedia component 1 Endoparasites according to animal groups Multimedia component 1
